# Factors influencing female physicians’ careers in the German-speaking part of Switzerland

**DOI:** 10.1038/s44401-026-00132-w

**Published:** 2026-08-05

**Authors:** Sara Maeder, Kerstin Vondruska, Norman Bitterlich, Petra Stute

**Affiliations:** 1 Faculty of Medicine, Doctoral Student, University of Bern, Bern, Switzerland; 2 Medical Women Switzerland, Physician, Zurich, Switzerland; 3 Freelance Statistician (Retired), Chemnitz, Germany; 4 Department of Obstetrics and Gynecology, Inselspital, University Hospital of Bern, Bern, Switzerland

**Keywords:** Health care, Health occupations, Medical research

## Abstract

Despite an increase in female physicians in Swiss hospitals, most executive positions are still held by male physicians. This study aimed to identify obstacles and institutional factors influencing female physicians’ careers and to evaluate ways to support their professional advancement. An online questionnaire on career obstacles, institutional support, and gender equality was distributed to female physicians employed at hospitals in German-speaking Switzerland. Statistical comparisons between subgroups were performed. The main obstacles reported were doubt in personal competence (39.8%) and lack of support from superiors (37.0%). Mentoring and connecting female physicians were identified as the most useful support options. Doubt in personal competence and a lack of institutional support hinder female physicians’ careers. Mentoring programs, flexible employment options, and improved childcare can support their advancement to executive positions.

## Introduction

Over the last decade, the number of female physicians in Switzerland has rapidly increased^1^. In 2022, 60% of students graduating from medical school in Switzerland were female^2^. In executive positions, the proportion of women declines with each successive rank, with only 18% of head physician positions held by women^1^. Explanations for this gender gap in executive positions are numerous. While factors such as hierarchical hospital structures, competitive academic environments, and limited support from superiors hinder all physicians striving for higher positions, female physicians face additional, gender-specific barriers. The most commonly cited factor is motherhood, with pregnancy, maternity leave, part-time work, and greater domestic responsibilities frequently identified as causes of career stagnation among female physicians^3–11^. Because childcare services in Switzerland are predominantly privately or semi-privately organized and costly, external childcare is frequently difficult to arrange. Furthermore, there is a distinct lack of options available, with 50,000 places lacking in 2011, especially for children under the age of four years and in rural environments.

Self-doubt appears to affect female physicians more than their male counterparts^6,7,12^, hindering career advancement. This confidence gap emerges in early education, with girls receiving the same grades as boys but showing less confidence in their performance, and continues into medical school and residency^12–15^.

In addition to motherhood and self-doubt, female physicians frequently report gender discrimination as a barrier to career advancement, encompassing unequal treatment in hiring, promotion, and daily workplace interactions^6,8,26^. These various obstacles often co-occur: the scarcity of part-time positions forces mothers to choose between career continuity and caregiving, while discriminatory workplace cultures reinforce the perception that leadership is reserved for those who conform to male working patterns. We therefore hypothesized that gender discrimination and lack of part-time positions are the most frequently reported career obstacles (H1) and that mothers are more likely to work in part-time positions and are less likely to occupy an executive position (H2).

Furthermore, female physicians receive less mentoring than male physicians^4,16^. Mentoring has been shown to raise career satisfaction and support professional development^17,18^.

Academic performance is another factor determining career progression. There are fewer women in academic medicine than men, often attributed to a lack of role models, frustrations with research due to lack of grants and mentoring, as well as a competitive, male-favoring environment, and poor work-life balance (especially for mothers)^19^. Female physicians publish fewer articles^20–22^ and are promoted to higher academic ranks at a slower pace than their male colleagues^22–24^. With less research activity than their male colleagues, female physicians are often deemed unsuitable candidates for executive positions, especially in university hospitals^25^.

This leads us to the hypothesis that support by superiors, mentoring, and professional associations are the most important tools to support female physicians’ career development (H3).

Many of these issues originate from deep-rooted structural and organizational gender discrimination. In gendered organizations, the implicit standard for an employee is a male full-time worker whose personal and family needs are met by others and who is fully committed to his job. Motherhood—encompassing pregnancy, birth, breastfeeding, and childcare—is therefore seen as grounds for exclusion, rendering women unable to meet the perceived demands of the position and confining them to lower-level roles with limited complexity and responsibility^26^.

With the health care system being known for its patriarchal structures, we hypothesized that especially young female physicians and female physicians employed at a larger hospital (e.g., university hospital) are affected by institutional gender inequalities (H4) and that female physicians claim that their employers are not doing enough to promote gender equality (H5).

According to surveys, ~10% of doctors working in acute care and up to 25% of doctors working in outpatient care will leave the profession in the next five years^27^. Reasons for female physicians leaving their profession include career burnout and incompatibility of work and family life^28,29^. With the additional burden of an ageing population, Switzerland cannot afford to lose female physicians^30^, as their departure would aggravate the existing shortage in health care workers, especially when considering the better patient outcomes and lower mortality rates among patients treated by female physicians^31–33^. Female researchers are more likely to include sex- and gender-related factors in disease-specific research, adding to the integration of gender into treatment recommendations^34,35^. Furthermore, women in executive positions have often outperformed their male counterparts, particularly regarding communication and employee support^36^. This led us to the hypothesis that female physicians with female superiors less frequently report gender discrimination as a career obstacle than female physicians with male superiors (H6).

Finally, we examined whether career obstacles differed between public and private hospitals. Because both sectors in Switzerland operate under similar labor regulations and medical training structures, and because the gendered organizational processes described by Acker are characteristic of hospital hierarchies in general rather than of a specific ownership model, we hypothesized that there would be no strong distinctions between public and private institutions (H7). Few studies have examined career obstacles for female physicians in Switzerland. Buddeberg-Fischer et al. showed gender-specific differences regarding workplace experiences in residency in German-speaking Switzerland^16^. They further reported lower employment rates, more part-time work, and slower career advancement among female physicians than male physicians 7 years after graduation^4^.

We were interested in learning about the experiences of female hospital physicians concerning gender discrimination and career development in German-speaking Switzerland. Focusing on this language region was due to economic reasons (the necessity of translating the questionnaire and responses) as well as a lack of connections to distribute the questionnaire in the French- and Italian-speaking parts of Switzerland, not due to assumed differences in respect to gender inequalities.

## Results

Subjects were deemed non-evaluable and excluded if the participant did not complete the survey or if the participant indicated in the comments that they worked exclusively as an attending physician with private practice privileges (Belegärztinnen), as this study focuses on hospital-employed physicians.

### Characteristics of the cohort

Table 1 presents the study sample’s characteristics. The median age was 36 years, with an IQR of [31, 43]. The median workload was 100% with an IQR of [80%, 100%]. 320 people (42.2%) had at least one child. Mothers had a lower workload with a median of 80% (vs physicians without children 100%, *p* < 0.001). Women in executive positions were not significantly less likely to have children than those in non-executive positions (mother in executive position *n* = 41, 65.1% vs mother in non-executive position *n* = 234, 58.0%; *p* = 0.385). Of all mothers, 120 (37.5%) stated that they were the main earner of the family; mothers in executive positions were more likely to be the main earner (mothers in EP *n* = 49, 30.6% vs mothers in NEP *n* = 71, 12.0%, *p* < 0.001).

**Table 1:** Sociodemographic characteristics and career-related factors

Overall: 754 respondents		
	#	%
Type of clinic		
*University hospital*	436	57.8
*Cantonal hospital*	172	22.8
*Regional hospital*	88	11.7
*Private clinic*	49	6.5
*other*	9	1.2
Position		
*Resident*	328	43.5
*Senior physician*	229	30.4
*Attending physician*	83	11
*Hospital specialist*	44	5.8
*Deputy senior physician*	37	4.9
*Head physician*	20	2.7
*Deputy head physician*	13	1.7
Specialization		
*Internal medicine*	184	24.4
*Surgical specialties*^a^	115	15.3
*Obstetrics & gynecology*	74	9.8
*Anesthesiology*	62	8.2
*Others*	319	42.3
Academic degree		
*Promotion*	536	71.1
*Habilitation*	36	4.8
*Associated professorship*	12	1.6
*Professorship*	12	1.6
*None of the above*	158	21
Workload		
*0–59%*	50	6.6
*60–79%*	119	15.8
*80–99%*	147	19.5
*100%*	438	58.1
In job-sharing		
*Yes*	31	4.1
Interested in job-sharing		
*Yes*	243	32.2
Age group		
*≤30*	140	18.6
*31–40*	366	48.5
*41–50*	171	22.7
*51–60*	68	9
*≥ 61*	9	1.2
Children		
*Yes*	320	42.2

^a^Surgical specialties = surgery, vascular surgery, hand surgery, cardiothoracic surgery, oral and maxillofacial surgery, neurosurgery, orthopedics, plastic and reconstructive surgery, traumatology, urology, pediatric surgery.

Overall, 36.3% of physicians were actively working on research projects, with physicians active in academic medicine having a higher workload (mean workload researchers 92% vs non-researchers 84%, *p* < 0.001) and being more likely to be employed at a university hospital (81.8% vs 44.2%, *p* < 0.001).

### Career obstacles

The most frequently reported career obstacle was doubt in personal competence, followed by lack of support from superiors (Table 2). Mothers’ most frequently reported career obstacles were a lack of or insufficient childcare and missing part-time positions. Doubt in personal competence was reported significantly less frequently by mothers than by female physicians without children (mothers *n* = 107, 33.4% vs no children *n* = 193, 44.5%; *p* = 0.003).

**Table 2:** Self-reported career obstacles

Overall: 754 answers	No Children (*n* = 434)		Mothers (*n* = 320)		*p*-value	EP (*n* = 160)		NEP (*n* = 594)		*p*-value	Overall	
	#	%	#	%		#	%	#	%		#	%
Doubt in personal competence	**193**	**44.5**	**107**	**33.4**	***0.003***	**51**	**31.9**	**249**	**41.9**	***0.023***	300	39.8
Lack of support from superiors	153	35.5	126	39.4	*0.253*	53	33.1	226	38	*0.269*	279	37
Missing part time positions	**84**	**19.4**	**104**	**32.5**	***<0.001***	**21**	**13.1**	**167**	**28.1**	***<0.001***	188	24.9
Lack of role models	103	23.7	75	23.4	*0.931*	41	25.6	137	23.1	*0.529*	178	23.6
Gender discrimination	97	22.4	79	24.7	*0.486*	43	26.9	133	22.4	*0.247*	176	23.3
Lack of/insufficient childcare	**39**	**9**	**128**	**40**	***<0.001***	40	25	127	21.4	*0.335*	167	22.1
Lack of support from professional associations	39	9	7	2.2	*0.001*	7	4.4	39	6.6	*0.357*	46	6.1
None	92	21.2	44	13.8	*0.01*	37	23.1	99	16.7	*0.064*	136	18

Career obstacles selected from a list. Noteworthy results (significant differences between groups with a *p*-value ***<*** 0.05) are marked in bold. *P*-values ***<*** 0.05 were considered significant.

Multiple answers were possible (max. 3).

*EP* executive position, *NEP* non-executive position.

Physicians active in research reported lack of support by superiors and lack of role models more frequently as an obstacle than physicians not active in research (43.1% vs 33.5%, *p* = 0.012, and 35.0% vs 17.1%, *p* < 0.001).

We found no significant differences in reported career hurdles in women working in public compared to private hospitals.

We further examined whether doubt in personal competence was associated with lack of support from superiors and lack of role models. In the overall sample, neither lack of support (adj. OR 0.80, 95% CI 0.58–1.09, *p* = 0.156) nor lack of role models (adj. OR 1.33, 95% CI 0.94–1.87, *p* = 0.111) were significantly associated with doubt in personal competence after age-adjustment. Age was the only significant predictor (adj. OR 0.97 per year, *p* = 0.004). However, when stratifying by parental status, lack of role models was significantly associated with doubt in personal competence among mothers (adj. OR 1.91, 95% CI 1.11–3.27, *p* = 0.019) but not among physicians without children (adj. OR 1.02, 95% CI 0.65–1.60, *p* = 0.94). Lack of support from superiors and lack of role models were significantly associated with each other (OR 1.97, 95% CI 1.40–2.77, *p* < 0.001).

### Institutional factors

Table 3 summarizes female physicians’ satisfaction with their employer’s commitment to gender equality. Most respondents were dissatisfied or somewhat dissatisfied, particularly employees at university hospitals and residents. Satisfaction levels did not differ significantly by age, parental status, or hospital privatization.

**Table 3:** Satisfaction with the employer’s effort to ensure gender equality

	Mothers *n* = 296			No children *n* = 425				EP *n* = 127			NEP *n* = 594				Overall *n* = 721		
	*n*	#	%	*n*	#	%	*p*-value	*n*	#	%	*n*	#	%	*p*-value	*n*	#	%
Type of clinic																	
*University hospital*	*178*	63	35.4	*253*	105	41.5	*1.000*	*54*	27	50.0	*377*	141	37.4	*0.104*	*431*	168	39.0
*Cantonal hospital*	*63*	37	58.7	*99*	54	54.5	*0.718*	***27***	**21**	**77.8**	***135***	**70**	**51.9**	***0.023***	*162*	91	56.2
*Regional hospital*	*29*	13	44.8	*49*	27	55.1	*1.000*	*18*	7	38.9	*60*	33	55.0	*0.352*	*78*	40	51.3
*Private clinic*	*22*	12	54.5	*20*	10	50.0	*0.520*	*25*	12	48.0	*17*	10	58.8	*0.708*	*42*	22	52.4
*other*	*4*	2	50.0	*4*	2	50.0	*0.238*	*3*	2	66.7	*5*	2	40.0	*1.000*	*8*	4	50.0
Age group (in years)																	
*≤30*	*11*	5	45.5	*129*	63	48.8	*1.000*	*0*	0	0.0	*140*	68	48.6	*---*	*140*	68	48.6
*31–40*	*146*	55	37.7	*219*	95	43.4	*0.328*	*18*	7	38.9	*347*	143	41.2	*1.000*	*365*	150	41.1
*41–50*	*103*	44	42.7	*52*	28	53.8	*0.254*	***68***	**39**	**57.4**	***87***	**33**	**37.9**	***0.025***	*155*	72	46.5
*51–60*	*31*	20	64.5	*21*	10	47.6	*0.355*	*36*	20	55.6	*16*	10	62.5	*0.870*	*52*	30	57.7
*≥61*	*5*	3	60	*4*	2	50.0	*1.000*	*5*	3	60.0	*4*	2	50.0	*1.000*	*9*	5	55.6
Gender of superior																	
*Male*	*259*	107	41.3	*381*	171	44.9	*0.416*	***107***	**57**	**53.3**	***533***	**221**	**41.5**	***0.032***	*640*	278	43.4
*Female*	*37*	20	54.1	*44*	27	61.4	*0.661*	*20*	12	60.0	*61*	35	57.4	*1.000*	*81*	47	58.0

Overview of physicians who stated “yes” or “rather yes” when asked if the employer is doing enough to ensure gender equality. Noteworthy results (significant differences between groups with a *p*-value < 0.05) are marked in bold. *P*-values < 0.05 were considered significant.

*EP* executive position, *NEP* non-executive position.

A large majority (672; 89.2%) agreed on the need for more female managers (yes and rather yes combined), with researchers (67.5% vs 53.8% among non-researchers answering yes, *p* = 0.003) and mothers being more likely to respond affirmatively (64.7% vs 54.4% in childless physicians answering yes, *p* = 0.030).

Women who reported gender discrimination as a career obstacle also reported lower satisfaction with employer efforts towards gender equality. They also identified a lack of support from superiors more frequently as a hurdle and viewed health policy measures, such as quotas, as critical for professional advancement (Table 4). Concerning career ambitions (strive for promotion within next 5 years), we found no differences between physicians who reported gender discrimination as a career obstacle and physicians who did not (promotion in next 5 years and gender discrimination as a career obstacle *n* = 79, 46.7% vs no gender discrimination reported *n* = 218, 39.5%; *p* = 0.113).

**Table 4:** Age-adjusted binary logistic regressions concerning gender discrimination

	Age-adjusted OR	95%-Confidence Intervals	*p*
Experienced lack of support by superiors as career hurdle (0 = no, 1 = yes)	3.038	1.99–4.64	*<0.001*
Of the opinion that health policy commitment (e.g., Quota Regulation) is a valuable support opportunity for professional associations (0 = no, 1 = yes)	2.271	1.51–3.42	*<0.001*
Of the opinion that the employer is doing enough to ensure gender equality (0 = no, 1 = yes)	0.276	0.17–0.43	*<0.001*
Female clinic leader (0 = no, 1 = yes)	0.442	0.21–0.93	*0.030*
Of the opinion that more women are needed in executive positions (0 = no, 1 = yes)	3.974	1.48–10.62	*0.006*

The answers of 176 people reporting gender discrimination as a career hurdle were compared to the 445 cases of people not reporting discrimination as a career obstacle using binary logistic regression analysis after age-adjustment. *P*-values < 0.05 were considered significant.

*OR* Odds Ratio.

### Support possibilities

To identify support strategies for female physicians, participants were asked to select the most helpful options from a list (Table 5).

**Table 5:** Support possibilities professional association

Overall: 754 answers	Mothers (*n* = 320)		No Children (*n* = 434)		*p*	EP (*n* = 160)		NEP (*n* = 594)		*p*	Overall	
	#	%	#	%		#	%	#	%		#	%
Health policy commitment (e.g., quota regulation)	118	36.9	152	35.0	*0.645*	57	35.6	213	35.9	*0.517*	270	35.8
Coaching (e.g., workshops)	132	41.3	141	32.5	*0.014*	63	39.4	210	35.4	*0.355*	273	36.2
Mentoring	222	69.4	309	71.2	*0.628*	114	71.3	417	70.2	*0.846*	531	70.4
Connecting female physicians	194	60.6	252	58.1	*0.500*	95	59.4	351	59.1	*1.000*	446	59.2
Support for research projects	95	29.7	137	31.6	*0.632*	48	30	184	31	*1.000*	232	30.8

Support options that could be offered by a professional association (selected from a list). *P*-values < 0.05 were considered significant.

Multiple answers were possible (max. 3).

*EP* executive position, *NEP* non-executive position.

When asked how the current position was attained, the majority of female physicians stated that they were hired after applying for the position. Promotion within the hospital was especially common among physicians in executive positions between the ages of 35–46 years (executive position 35–46 y *n* = 38, 60.3% vs. non-executive position 35–46 y *n* = 106, 39.4%; *p* = 0.012).

The most frequently chosen supporting factors to achieve the current position were professional qualifications (*n* = 710, 94.2%, with 754 participants overall) and encouragement by superiors (*n* = 366, 48.5%, with 754 participants overall).

## Discussion

This study provides a comprehensive overview of factors influencing the career progression of female physicians in the German-speaking part of Switzerland. Our findings largely confirm our initial hypotheses, though with notable nuances.

Regarding our specific study objectives:

- Career Obstacles (H1): Partially confirmed. Doubt in personal competence and gender discrimination were major barriers, though doubt in competence (39.8%) slightly outranked discrimination (23.3%).
- Motherhood (H2): Partially confirmed. While mothers work significantly lower percentages, our data did not support the hypothesis that mothers are less likely to hold executive positions compared to non-mothers (*p* = 0.385).
- Support (H3): Confirmed. Mentoring and networking were identified as the primary support mechanisms.
- Institutional Gender Inequalities (H4): Confirmed. Satisfaction with employer efforts toward gender equality was lowest among university hospital employees (39.0%) compared to cantonal (56.2%), regional (51.3%), and private hospitals (52.4%). However, the hypothesized age effect was not clearly supported: physicians aged ≤30 reported higher satisfaction (48.6%) than those aged 31–40 (41.1%), suggesting that dissatisfaction may peak during career stages where advancement decisions are made rather than simply among younger physicians.
- Employer Efforts (H5): Confirmed. The majority of respondents were dissatisfied with their employer’s commitment to gender equality, and 89.2% agreed that more female managers are needed.
- Female Superiors (H6): Confirmed. Having a female clinic leader was associated with significantly lower reported gender discrimination (adj. OR 0.44, 95% CI 0.21–0.93, *p* = 0.030).
- Public vs. Private Hospitals (H7): Confirmed. No significant differences in reported career obstacles were found between public and private hospitals.

We found doubt in personal competence and gender discrimination to be the most significant career obstacles, with discrimination reported particularly frequently by physicians with male leaders. Mothers struggle with finding a work-life balance and work fewer hours. However, they seem to be less hindered by doubt in personal competence. We interpret this counter-intuitive finding as a potential ‘survivor bias’ or resilience effect: female physicians who manage to remain in the demanding hospital sector while raising children may possess higher baseline resilience. Those with higher self-doubt may have already left the hospital workforce for outpatient care or other fields.

The physicians were dissatisfied with the employers’ efforts to ensure gender equality; having female superiors was associated with higher satisfaction levels. Mentoring and networking were rated as the most useful tools to support female physicians. Younger physicians in executive positions were more likely to be promoted within the employing clinic and profited from endorsement from superiors. Female physicians in German-speaking Switzerland, therefore, face similar challenges and structural issues to those reported by their colleagues abroad. To support female physicians in their career advancement, two approaches need to be discussed: 1) utilizing and strengthening the female physicians’ resources with mentoring programs and coaching, and 2) structural changes in the employing institutions, especially concerning work-life balance, as well as in the educational system.

Mentoring was rated as one of the most useful measures to support career development. Mentoring has consistently been associated with enhanced career preparation and professional development^7,17,18,37,38^. Female physicians are still underrepresented in mentoring programs^4,39^. Thus, hospitals and professional associations offering mentoring programs that address doubt in personal competence and career challenges can support female physicians in creating sustainable pathways for growth across specialties and career stages.

Concerning academic medicine, several interventions to support female researchers have been proposed: Allan et al. worked out the following strategies: stay interviews (where the individual’s needs to remain at the current institution are identified), repetitive analyses of faculty metrics (amongst others: pay, promotions, publications, awards) to identify gaps and measure the effectiveness of interventions, implementing best practices for paid parental leave and return-to-work policies, inviting female researchers to publish with senior faculty, and avoiding gender-based bias in faculty evaluation^40^. A successful example for extensive structural interventions to recruit and retain more women in academic medicine is the Women in Medicine and Health Science (WIMHS) program that was established in the University of California Davis School of Medicine, resulting in an increase in the number and percentage of female faculty and department chairs with a low departure rate in female faculty. The WIMHS program covers promoting women’s leadership in different areas, as well as sustainable institutional strategies to enhance equity. It further involves data collection and analyses concerning gender equity; mentoring and career development programs, regular networking events, and close collaboration with medical schools^41^, which are inevitable, as female students and graduates show less interest in academic medicine than their male colleagues^42,43^. Essential is furthermore the adaptation of work hours to be able to also partake in family life^44^.

When revisiting the topic of gendered organizations, where the ideal worker is implicitly male—fully available, unencumbered by family responsibilities—we can see how the scarcity of female leaders in hospitals is a product of these gendered organizational processes, and in turn perpetuates them: without visible role models who have advanced despite non-conforming career trajectories, mothers in particular internalize doubt about whether advancement is achievable for them. This is highlighted by our finding that lack of role models predicts self-doubt specifically among mothers (adj. OR 1.91, *p* = 0.019).

Ways must be found to dismantle gendered employment structures and create space for both women and men to balance work and family responsibilities. Such changes benefit not only women but offer all employees a better balance of work and family, a demand that (also in men) has become increasingly apparent in recent years^45,46^. Possible structural changes can be divided into institutional and societal categories. Health care institutions should consider four pillars to improve gender equality and thereby retain (female) physicians in their profession: 1) recruitment and promotion, where a balanced gender distribution should be sought at all levels and objective selection and performance assessments have to be implemented; 2) career development, with institutions nurturing mentoring programs, career counseling, and sponsorships, where mentors use their influence to offer support for promotions and advancement opportunities; 3) work-life compatibility, where health care institutions can support physicians with implementing on-site childcare services, offering part-time positions (including for leadership positions), and supporting alternative leadership structures such as job/top sharing; and 4) management and culture, with implementation of regular data collection regarding gender equality and mandatory bias training for leadership and selection committees^9,47–49^. Each of these pillars directly addresses the gendered organizational processes described by Acker^26^. Objective recruitment criteria challenge the implicit male standard of the “ideal worker,” while mentoring and sponsorship counteract the exclusion of women from informal advancement networks. Flexible work arrangements and on-site childcare disrupt the assumption that full-time availability is a prerequisite for leadership. Finally, systematic monitoring of gender equity ensures that these processes are made visible and accountable rather than left to reproduce themselves unchecked.

When focusing on societal factors, so-called child penalties (parenthood leading to gaps in earnings, fewer working hours, and labor force participation) still mainly concern mothers^3–5,8–11^. Therefore, comprehensive and inexpensive childcare services that are also available for young children, as well as generous parental leave, are essential for female physicians to remain and succeed in their profession, as shown by Eydal et al.^50^.

Furthermore, gender sorting processes need to be addressed. Gender sorting means separating individuals into groups based on gender due to social norms and stereotypes^51^. These processes often begin in primary school and continue to management levels: Qian et al. argue that high-achieving female students are often encouraged to enter feminine-typed college majors with fewer chances to occupy leadership roles, whereas high-achieving male students are steered into masculine-typed college majors, such as science, technology, engineering, and mathematics (STEM) or finance fields, with more opportunities to advance in executive positions^8^. The organizational sorting process then continues, leading to men being placed in managerial positions and women in low-authority roles. Recommended interventions are changes in the organization of educational systems, limiting the influence of gender stereotypes on students’ trajectories, refraining from gender labeling of fields, and building communities for women in STEM, as well as reducing biases in hiring and promotion^8,52–54^.

This study provides insights into perceived career obstacles, the influence of superiors’ gender on institutional satisfaction, and the persistent struggle of mothers to balance work and family life. However, several opportunities for further research remain. First, incorporating the perspectives of male physicians would enable a comprehensive comparison of gender-specific experiences and attitudes, thereby enriching the understanding of gender dynamics in medical careers. Second, extending the study to include the French- and Italian-speaking regions of Switzerland could provide a broader perspective on regional differences in gender equality and career barriers. Widening the scope to include physicians who have left direct patient care could add valuable information about factors leading to doctors’ burnout. For questions on career obstacles and support options, respondents could select a maximum of three answers, meaning that reported frequencies reflect prioritization rather than exhaustive reporting. This constraint precludes certain multivariate clustering techniques but does not affect group comparisons or regression analyses, where it applies symmetrically across groups. However, the descriptive power remains high due to the sample size. Lastly, future studies should explore whether the observed trends disproportionately reflect negative experiences, as it is possible that women with unfavorable circumstances were more likely to participate in the research. By addressing these areas, future research can further clarify the nuanced dynamics of gender inequality in medical careers and contribute to the development of effective, evidence-based interventions.

## Methods

### Study design

The study was conducted as an anonymous, cross-sectional online survey of female physicians working in the German-speaking part of Switzerland. The Cantonal Ethics Committee of Bern approved the study protocol (BASEG-Nr. Req-2019-01274, approved by Prof. Dr. med. Ch. Seiler and Dr. sc. nat. Dorothy Pfiffner) and did not classify the study as research under the Human Research Act. The research, and especially the handling of the data, has been performed in accordance with the Declaration of Helsinki.

### Questionnaire

We used a purpose-designed, non-validated questionnaire (Supplementary File 1). The target population was female hospital physicians working in the German-speaking part of Switzerland. Participants were informed about the purpose of the study, the survey duration, and the data anonymization. They were further made aware that by completing the questionnaire, they consented to the processing of their data.

The questionnaire was developed in several steps based on a literature review, personal experiences among the study group, and input from members of a professional association. It was reviewed by the statistician (NB) to test for statistical evaluability and adapted accordingly. After a final review by all co-authors, the definitive questionnaire comprised five domains: 1) medical specialization and current job position, 2) career development and professional/academic ambitions, 3) experienced career obstacles/support factors, 4) participant’s opinion on gender equality, perceived employers’ efforts to support female physicians, and 5) sociodemographic information and research activities. For some questions, multiple answers were possible. Questions were asked or left out based on previous answers (e.g., when a participant stated not to have any children, they were not asked the number of children). The maximum of questions to answer was 31 items. Participants were able to correct their answers if needed. The survey was programmed with the online tool umfrage-online.com, a secure website with an encrypted database, and tested by study group members before being deployed. Our survey was open (not password-protected) and put online between March and August 2020. A selection of hospitals in the German-speaking part of Switzerland was contacted and asked to distribute the link to the survey to employed female physicians. All university and cantonal hospitals and at least one regional hospital per German-speaking canton were contacted.

Furthermore, a private clinic group was asked to distribute the survey. The participation of hospitals and employees was entirely voluntary, and there was no advertisement for the survey or incentives offered. We did not directly contact the participants and do not know how many physicians were sent the link to the questionnaire. Therefore, a participation rate cannot be given.

No specific mechanisms (such as Cookies or IP checks) were used to prevent multiple entries from the same user.

### Participants

The inclusion criteria were female gender, a completed medical degree, and employment at a public or private hospital in the German-speaking part of Switzerland. The exclusion criteria were unfinished questionnaires and employment as an occupational physician, as we focused on career hurdles in a hospital context.

### Study objectives

#### Self-reported career obstacles

We asked the participants about perceived career obstacles and gave them a selection of possible answers. A maximum of 3 options could be chosen. We hypothesized that gender discrimination would be the most frequently chosen career obstacle and that subgroups (mothers, physicians active in research) would report different obstacles.

#### Institutional factors influencing the work experience

The workplace and superiors can actively support or neglect the needs of female physicians. We were interested in finding differences in workplace properties and satisfaction in the workplace’s commitment to gender equality.

#### Support possibilities

To find possibilities for professional associations to support female physicians better, we gave the participants a selection of possible support options, again with a maximum of 3 choices.

### Bias

Our study could be subject to selection bias (especially volunteer bias), as physicians aware of or personally affected by gender discrimination could have participated in the survey to draw attention to their grievances.

### Statistical methods

Statistical analyses were conducted using IBM SPSS Statistics Premium Campus Edition 26 (Armonk, NY, USA).

We summarized categorical variables as counts and percentages and compared them using Chi-square or Fisher’s exact tests where expected counts <5. Continuous variables were reported as mean ± standard deviation (SD). Non-normally distributed continuous variables (age and workload) were summarized as medians with interquartile ranges (IQR) and compared between groups using the Kruskal–Wallis test to assess differences in distributions. To characterize the subjective assessment of “gender discrimination”, we only analyzed participants who affirmed gender discrimination (*n* = 176) or who did not indicate gender discrimination but gave a maximum of two answers regarding barriers in their career (i.e., they did not exhaust the answer limit of three answers and deliberately left out gender discrimination, *n* = 445). After adjusting for age, as this was the only covariate to meaningfully reduce bias without drastically increasing the complexity of the model, we calculated binary logistic regressions to establish links between gender discrimination and other factors influencing the careers and personal attitudes of the respondents.

*P*-values were computed to evaluate statistical significance, with *p* < 0.05 considered significant.

To explore whether doubt in personal competence was associated with institutional factors, we calculated age-adjusted binary logistic regressions with doubt in personal competence as the outcome, stratified by parental status. Pairwise associations between binary career obstacles were assessed using odds ratios with 95% confidence intervals.

Concerning subgroup analyses according to positions, we classified attending physicians (Leitende Ärztinnen), hospital specialists (Spitalfachärztinnen), deputy head physicians (Stellvertretende Chefärztinnen), and head physicians (Chefärztinnen) as physicians in executive job positions.

## Supplementary information

Supplementary Information

## Data Availability

The datasets generated and analyzed during the current study are not publicly available due to endangerment of the participants’ privacy, but are available from the corresponding author on reasonable request.
