# Supplementary material for: Factors influencing female physicians’ careers in the German-speaking part of Switzerland
Source: Npj Health Syst. 2026 Aug 5;3:71. doi: 10.1038/s44401-026-00132-w (PMC13439069; doi:10.1038/s44401-026-00132-w)
Supplement: Supplementary file 1 [file 44401_2026_132_MOESM1_ESM.docx]

**Questionnaire**

Dear colleague

It is well known that there are fewer women in leadership positions than men, this is also true in the healthcare sector.

This survey is launched by medical women switzerland mws, a professional association that has been advocating for Swiss female physicians and medical students for over 90 years.

The goal of the survey is to find factors that influence the unequal gender distribution in leadership positions and to show how professional associations like mws can advocate for more equality.

Answering the questions will take about 5 min. Of course, your information will be treated anonymously and confidentially.

Thank you very much for your feedback!

<https://www.medicalwomen.ch/de/home/>

1. **What type of hospital do you work in?**

Regional Hospital

Cantonal hospital

University Hospital

Private clinic

Other

2. **What is your specialization?**
   (chose from drop-down menu with all SIWF disciplines listed)
3. **What is your position?**
   Resident

Deputy senior physician

Senior physician

Chief physician

Deputy chief physician

Chief physician

Hospital specialist

4. **If resident: Are you employed on a limited basis?**

Unlimited
Limited to 1 year

Limited to 2 years

Limited to 3 years

Limited to other time period

5. **If resident:** **Are you pursuing a specialist title?**

Yes / No

6. **What is your workload (in percent)?**

Enter number

7. **How did you obtain this position?**

Hired after applying for job posting

Hired through networking (e.g. referral to position by supervisor, colleagues)

Promotion within the clinic

Other

8. **For how many years have you held this position?**

Enter number

9. **Do you have enough time to perform all the duties your position entails? (E.g. patient care, teaching, research, leadership roles, continuing education)**
   Yes / No
10. **Do you plan to remain in your current position for the next 5 years?**
    Yes / No
11. **If No: Do you plan to,**
    to take a higher position?
    to become self-employed?
    to leave the health care sector?
    to retire?
    Other
12. **Do you work in job sharing (a position is divided between at least 2 employees, who can individually determine the working hours)?**

Yes / No

13. **Would you like to work in job sharing?**

Yes / No

14. **Who or what helped you to reach your current position? (Please select the three most important points for you)**

Professional qualifications

Encouragement by superiors

Professional association

Female networks

Personal relationships (family, partnership, friends)

Personal experiences (stay abroad, research)

Female gender

15. **What hurdles did you face to achieve your position?**

**(Please select the three most important to you)**

Gender discrimination

Lack of part-time positions

Lack of/insufficient childcare

Lack of support from superiors

Lack of support from professional association

Lack of role models

Doubts in personal competence

16. **Is it important to you that more women occupy management positions?**

Yes

Rather yes

Rather no

No

17. **How can a professional association like mws support women's hospital careers?**

**(Please select the three most important points for you)**

Health policy commitment (e.g. advocacy for quota regulation)

Coaching (e.g. workshops)

Mentoring (individual support by a specialist)

Connecting female physicians

Support for research projects

18. **Do you think your employer is doing enough to promote female physicians?**

Yes

Rather yes

Rather no

No

19. **What is the gender of your clinic director?**

Female / Male

20. **What is your age (in years)?**

Enter number

21. **How much do you earn gross per month (in CHF)?**

<5'000 CHF

5'000-10'000 CHF

>10'000 CHF

22. **Do you receive a pool participation?**

Yes / No

23. **Are** **you in a relationship?**

Yes / No

24. **If yes: Does your partner work full time?**

Yes / No

25. **Do you have children?**

Yes / No

26. **If yes: Age of children (in years)**

(Multiple answers possible)

Enter number

27. **If yes: Are you the main earner of your family?**

Yes / No

28. **Do your parents have an academic degree (university or college)?**

No

Mother

Father

both

29. **What is your highest academic degree?**

Doctorate

Habilitation

Associated professorship

Professorship

30. **Which academic degree are you aiming for?**

Doctorate

Habilitation

Associated professorship

Professorship

31. **Are you active in research?**
    Yes / No
32. **If no: what are the reasons for not being active in research?**
    Lack of interest
    Lack of possibilities

Lack of time
No support from employer
Not compatible with social life
Other

33. **Feedback to the survey**

Enter text

Thank you for your answers!
